# Climate change in Türkiye and its impact on oncology nurses

**DOI:** 10.3332/ecancer.2023.1623

**Published:** 2023-11-09

**Authors:** Afşin Ahmet Kaya, Aydanur Aydin, Gülcan Bağcivan

**Affiliations:** 1 Emergency Aid and Disaster Management Department, Health Science Faculty, Ondokuz Mayıs University, 55139 Samsun, Türkiye; 2 Department of Nursing, Health Science Faculty, Gumushane University, 29100 Gumushane, Türkiye; 3 School of Nursing, Koc University, 34450 Istanbul, Türkiye

**Keywords:** climate change, oncology, cancer, nurse, Türkiye

## Abstract

Climate change threatens human life and health by negatively affecting the basic components of health such as clean air, safe drinking water, nutritious food supply, and safe shelter. Türkiye is a country that is largely exposed to climate change with its cosmopolitan location, which is a bridge between Asia and Europe. Due to climate change, serious effects are seen in all sectors from energy to agriculture, from the economy to health. Climate change is defined as the most important global health threat of the next century, and the problems it brings are seen as the most important pressure factor for the life opportunities of future generations. Food and fresh water availability, rising sea levels, abnormal weather events, migration, and diseases are thought to affect human health. A multidisciplinary approach is required to adapt to climate change’s health effects and reduce its negative health effects. In addition, it is predicted that diseases that are about to disappear will re-emerge and become threats. It is thought that as a result of the contamination of food and water resources with the changing ecosystem, some infections will increase and society will face them. Nurses are both affected by climate change and its effects on public health. Nurses who remain in this situation have a great role in providing effective awareness for raising public awareness. In recent years, oncology clinics have not been affected by fire, earthquakes, and pandemics in our country. In this respect, it can be stated that the health service in Turkish oncology clinics is carried out uninterruptedly and by experts in the field. We learned during the pandemic that the whole world is unprepared for the future effects of climate change. In this direction, nurses should think about solutions for the problems related to climate change in the future.

## Introduction 

The consumption society has begun to end the limited resources in our common living space. Therefore, technological developments have started to increase to respond to human needs and make life easier. These technological developments, which include important steps for humanity, have had beneficial outputs. However, in recent years, it has been seen that uncontrolled industrialisation has caused some balance confusion. For example, changes in climate parameters are evidence of this confusion [2]. It is seen in the statistical reports published by the World Meteorological Organization, the International Energy Agency, the National Oceanic and Atmospheric Administration, and the Türkiye Meteorology General Directorate (MGM), that parameters such as temperature, precipitation, humidity, and wind are on a different course compared to their current averages [2, 3]. The negative change in the climate phenomenon, which has a critical importance for the survival of humans and other living species, is now felt by living things seriously. The reasons responsible for this negative change are divided into two parts. These man-made and natural causes are as follows:

Natural causes are the Milanković cycle [2], sunspots, continental drift, volcanic eruptions, the long-term flattening of the earth’s orbit around the sun, and losses of Antarctic and arctic glaciers. Human causes are also the industrial revolution, increasing greenhouse gas emissions, especially carbon dioxide emissions, population growth and unplanned urbanisation, destruction of forest areas, increasing use of fossil fuels, increasing the amount of carbon footprint, chemical pollution, and increasing amount of waste.

When we look at the causes of climate change given as substances, it is known that natural causes occur in the long term. The reason for the situation experienced in recent years is climate change caused by anthropogenic (resulting from the influence of human beings on nature) reasons. Climate change, the effects of which are increasing all over the world, has become a serious problem in our country [2, 3].

## Climate change in Türkiye

It is striking that the importance given to climate-related studies in Türkiye has started to be given importance politically in recent years. Türkiye has signed important steps for the fight against climate change in the international arena. The United Nations (UN) Framework Convention was signed in 2004 and the Kyoto Protocol in 2009. The Paris Climate Agreement, which includes important sanctions on climate change, was signed in 2016; however, it passed the approval of the Türkiye Grand National Assembly in 2021. The ratification process was prolonged because the conditions contained in this agreement caused financial difficulties for Türkiye. The Ministry of Environment and Urbanization, on the other hand, entered a structural revision process by taking the name of the Ministry of Environment, Urbanization, and Climate Change after the changes were made. ‘Türkiye Climate Change Strategy/CCS (2010–2023)’, which includes planning and future steps within the scope of combating climate change, is Türkiye’s first climate change strategy document. Later, the document ‘Climate Change Action Plan of the Republic of Türkiye/CCAP (2011–2023)’ was published. Finally, ‘Türkiye’s Climate Change Adaptation Strategy and Action Plan (2011–2023)’ has been published [15]. The common features of these documents are that they deal with important issues such as combating the effects of climate change, reducing greenhouse gas emissions, and adapting to climate change [2].

The Intergovernmental Panel on Climate Change (IPCC) has included striking information about the geography of Türkiye in its reports. The UN Secretary António Guterres has made it clear that the 1.5°C warming threshold will be exceeded in 20 years, and this is clearly due to human-induced effects. According to the sixth evaluation report, it is mentioned that the intensity and severity of temperatures in Europe will increase. This situation will also negatively affect Türkiye. The report also highlights the drought that will be caused by extreme temperatures that will occur in the Mediterranean region. Forest fires in 2021 are also thought to be related to climate [8, 9, 2, 3]

According to the results of the studies carried out by the Türkiye Statistical Institute, the total greenhouse gas emissions of the greenhouse gas rates, which are an important cause of climate change, in 2020 have been calculated as 523.9 million tons of CO_2_ equivalent, increasing by 3.1% compared to the previous year [7]. While the concentration of CO_2_ gas was around 219.7 ppm in 1990, this rate reached 524 ppm in 2020. The sector that causes the most greenhouse gas emissions in Türkiye is the energy sector [7]. Türkiye has an increasing trend in terms of the temperature curve. When we look at the situation in recent years, it is seen that the average temperature in 2021 is 1.4°C higher than the average from 1981 to 2010. This rate is 1°C higher than 1991–2020; 2021 is the distinction of being the 4th hottest year [5]. Irregularity and unclear estimates of the precipitation regime are also present in the IPCC reports. The results of the analysis for Türkiye also show the same situation. Total precipitation in 2021 was 524.8 mm. This rate is 9% less than the rate of 1981–2010 (574 mm) and 1991–2020 (573.4) [5]. Due to climate change, the number of extreme events is also increasing. At the point where the disaster resistance of society is insufficient, these events emerge as disasters. 2021 has been recorded as the year with the highest number of extraordinary events for Türkiye [5]. It was observed that most storms and tornadoes occurred in the events that occurred in 2021. This is in line with the IPCC 6th assessment report. Then hail, heavy snow, lightning, frost, avalanche, and forest fires are the types of meteorological disasters that have the highest percentage of incidents [5]. The data of the last 10 years of the General Directorate of Meteorology indicate that there is basic evidence of climate change in parameters such as temperature, precipitation, and humidity.

## Effects of climate change on Türkiye

Researchers and reports from international institutions show that; the increase in temperature caused by climate change will seriously affect Türkiye [2]. It is highly probable that there will be an increase in temperature in all regions of the country, and that these increases will continue in summer and winter, and the irregularities in the precipitation regime will occur according to the 2030 scenario. It has been observed that there will be an increase in the annual average temperature of Türkiye between 2.5°C and 4°C. Although there is an increase in precipitation in the eastern part of North Anatolia, precipitation is expected to decrease throughout Türkiye. All these will bring along a serious decrease in our country’s water resources, drought and desertification, loss of agricultural productivity, an increase in the number and impact of forest fires, and loss of biodiversity. Different regions of Türkiye will be affected by climate change differently and to varying extents. For example, arid and semi-arid regions such as South East and Central Anatolia, which are under the threat of desertification rather than temperature increase, and semi-humid Aegean and Mediterranean regions that do not have sufficient water will be more affected. Water resources in arid and semi-arid areas, especially in cities, will add to the problems and the need for drinking water will increase [8, 9, 2, 3].

There are warnings that climate change may cause adverse effects in terms of agriculture, forestry, and water resources (this is redundant). According to researchers, climatic zones may shift hundreds of kilometers from the equator to the poles, as in the geological past of the earth, and as a result, Türkiye may fall under the influence of the hot and arid climate zone prevailing in the Middle East and North Africa today. There are warnings that climate change may cause adverse effects in terms of agriculture, forestry, and water resources. According to researchers, climatic zones may shift hundreds of kilometers from the equator to the poles, as in the geological past of the earth, and as a result, Türkiye may fall under the influence of the hot and arid climate zone prevailing in the Middle East and North Africa today.

## Effects of climate change on health

Climate change has initiated detrimental impacts on human health. Extensive research has established that climate change is associated with an upsurge in cardiovascular, respiratory, and circulatory system disorders among individuals [10]. It is also another negative effect of climate change that is known to harm the psychological health of individuals [3, 4]. Depending on climate change, according to MGM data, there has been a serious increase in the number of meteorological disasters in the last 10 years. The increase in storms and forest fires is proof of this. As a result of the increasing frequency of climate-related disasters, the resilience of societies will decrease, and if measures are not taken, it can be expected that Türkiye will be more vulnerable in the fight against climate change in the future [5, 8, 9, 2, 3]

Climate change has direct or indirect effects on health. Direct effects; temperature extremes, heat/cold waves, hurricanes, storms, floods, and fires; indirect effects; vectorial diseases, infections, epidemics, water, and food-borne diseases, air pollution and respiratory diseases, stratospheric ozone depletion and UV radiation, allergic diseases, and field dust [12, 13]. Climate change has direct effects on some infectious diseases. Both direct and indirect effects of climate change are seen in Türkiye. According to Karabacak *et al* [11], regions with local forest fire risk were revealed in the province of Antalya. Accordingly, while all of the forests of Antalya province are at risk of fire, only 4% of them are at low risk, while 76% are at high risk in terms of forest fires [11]. A large fire occurred in Antalya in 2021 and many forests were affected by this fire [14]. This is one of the direct effects of climate change in our country.

One of the indirect effects of climate change in our country is the increase in vectorial diseases due to the increase in the number of immigrants from Asia. It is estimated that over 2 million Syrians came to Türkiye due to the civil war after April 2011 [6]. Many infections threaten immigrants, including food and waterborne diarrhea (typhoid and cholera), vector (plague and malaria), contact (Hepatitis A, shigellosis), and respiratory infections (measles and flu). The most common diseases are diarrheal diseases (including cholera and bacillary dysentery), measles, acute lower respiratory tract infections, and malaria [1].

## Effects of climate change on oncology nurses

Although climate change-related extreme environmental events are very common in Türkiye, we do not have any published data regarding how oncology nurses or oncology care services were affected by these climate change events in Türkiye. However, from the perspective of our experiences and interviews with our colleagues who are working in cities where these climate change-related extreme environmental events occurred, we know that oncology care was not stopped and was not affected negatively because of these events. Oncology nurses continued to provide care under the circumstances of these extreme situations since disruption in health service delivery did not occur. Even though the health care services for cancer patients have not been cut, from our experiences we know that all health care providers and patients were negatively affected emotionally like all other people. There are not enough studies to obtain detailed information. This situation shows us that new research needs to be done.

The effects of climate change seen in Türkiye in the last 5 years can be expressed as fire, earthquake, pandemic, and migration as stated above. When all the above-mentioned events occurred, nurse support from other departments was needed, especially for the emergency services in the relevant regions. Especially in the last pandemic period, nurses from many departments have been assigned to work in pandemic clinics. However, oncology nurses generally remained in their fields of work. Oncology nurses are assigned to different units from their units as a last resort so that oncology nursing requires a specialisation and service delivery in the field of oncology is not disrupted. For example, during the fire in Antalya or an earthquake in Van, oncology nurses were not assigned to work in the emergency department or in another department.

Although cancer care was not affected negatively during climate change-related extreme environmental events such as fire, and flood, it was disrupted due to a devastating earthquake that hit the eastern part of Türkiye including ten cities in February 2023. Almost 50,000 people died from earthquakes. The earthquakes have had a major effect on Türkiye’s healthcare system, causing damage to hospitals and health clinics in the affected areas, resulting in a shortage of medical facilities for injured and displaced people. Furthermore, the loss of power, water, and communication systems has complicated the healthcare system’s response efforts. Hospitalised patients who live in earthquake areas were transferred to other cities. For cancer patients who receive active chemotherapy or other treatment, cancer professional societies and some of the private hospitals offered transportation and treatment in big cities for free. The Government and NGOs have been continuing to meet the needs of housing for the victims but it is not desired level yet. It will not be a surprise that these negative experiences will cause problems such as delayed early diagnosis, and treatment and inability to receive adequate supportive care, as well as general health problems in the future. The loss of hospitals and weakened infrastructure is a long-term problem that will require considerable investment.

Although these problems seem manageable today, they are full of unknowns on how they will reflect the increasing effects of climate change. The pandemic, which is a problem we have yet to overcome, may have given us practice on how to manage problems in a health crisis that may occur. However, the expected drought in the future and the migrations that may occur due to this will include the management of new epidemic diseases and the problems that may come with it. In this direction, suggestions will be needed to manage the consequences of climate change that may occur worldwide and chronic health problems such as cancer.

## Suggestions

Assessing patients’ environmental exposures: Nurses can evaluate the environmental exposures of cancer patients and provide recommendations to reduce their risks. For example, they may suggest strategies such as reducing patients’ exposure to sunlight, promoting healthy eating habits, and advocating for access to clean drinking water.

Responding to patients’ psychosocial needs: Nurses can improve the emotional and social well-being of cancer patients by responding to their psychosocial needs. This can help patients cope with the effects of climate change and adapt to their treatment.

Developing climate change adaptation strategies: Nurses can assist cancer patients in developing strategies to adapt to climate change. This may include preparing patients for potential environmental risks, creating contingency plans, and promoting environmental protection measures to reduce risks.

Increasing environmental health awareness: Nurses can help protect public health by raising awareness about the impacts of climate change and environmental health risks. This is an essential step in addressing global threats like climate change and can empower cancer patients and the public to take action to safeguard their health.

The prompt response to the disaster by the country’s cancer communities and the transfer of cancer patients to qualified units in nearby provinces highlight the importance of disaster preparedness and response planning in healthcare. By having a well-coordinated plan in place, healthcare providers can respond quickly and effectively to natural disasters, ensuring that critical patient care is maintained even in the face of significant challenges. This approach can be particularly important for vulnerable patient populations, such as cancer patients, who may require specialised care and treatment.

## Conclusion

In conclusion, this example underscores the importance of disaster preparedness and response planning in healthcare and the critical role that healthcare providers can play in ensuring that patient care is maintained in the face of challenging circumstances. Nurses can play a vital role in helping cancer patients manage the challenges of climate change and promoting environmental health awareness.
